# Loss and Retention of RNA Interference in Fungi and Parasites

**DOI:** 10.1371/journal.ppat.1003089

**Published:** 2013-01-24

**Authors:** Francisco E. Nicolás, Santiago Torres-Martínez, Rosa M. Ruiz-Vázquez

**Affiliations:** 1 Departamento de Genética y Microbiología, Facultad de Biología, Universidad de Murcia, Murcia, Spain; 2 Regional Campus of International Excellence “Campus Mare Nostrum”, Murcia, Spain; Duke University Medical Center, United States of America

## The RNAi Pathway

RNA interference (RNAi) or RNA silencing is a gene regulatory system, widely conserved in eukaryotes, that represses gene expression through a homology-dependent mechanism. This repressive effect is mediated by small non-coding RNAs (sRNAs) of about 20–30 nucleotides, derived from double-stranded RNA (dsRNA) precursors that are recognized and processed by the RNaseIII Dicer. These sRNAs are loaded into an RNA-induced silencing complex (RISC), where the Argonaute protein plays a main role. Upon loading, the sRNAs selectively guide RISC to the target RNAs, causing their degradation or preventing their translation. In certain organisms, including fungi and parasitic protozoa, the silencing mechanism requires RNA-dependent RNA polymerases (RdRPs) to generate dsRNA from single-stranded RNA (ssRNA) or to amplify sRNA signals [1], [2]. Originally described as a defense mechanism against invasive nucleic acids and viruses, RNAi and related pathways play many fundamental roles in metazoans, including regulation of mRNA accumulation and translation, chromatin silencing, programmed DNA rearrangements, and genome surveillance.

## RNAi in Fungi and Parasites

An RNAi-related phenomenon called “quelling” was first described in fungi in the ascomycete *Neurospora crassa* [3]. Pioneering genetic dissection of silencing in this fungus [4] allowed the identification of the main genes involved and the characterization of the pathway and has been instrumental for further work in the field [1]. The best-understood function of the fungal RNAi machinery is to build pericentric heterochromatin in *Schizosaccharomyces pombe*, where RNAi is required for proper centromere function [5], [6]. In this process, specific histone modifications at centromeric regions are triggered by RITS (RNA-induced transcriptional silencing) complexes containing an Argonaute protein bound to centromeric siRNAs. These siRNAs are generated from centromeric repeat transcripts with the participation of RdRP and Dicer proteins. The specialized histone modifications are in turn responsible for the maintenance of the transcriptionally silent status of the heterochromatin [6]. Besides that, endogenous small RNAs (esRNAs) with putative regulatory functions have been identified in fungi (see below for more details), suggesting a functional diversification of RNAi pathways in these organisms.

The trypanosomatid protozoan *Trypanosoma brucei* was one of the first organisms in which RNAi was discovered [7]. Since then, this mechanism has been extensively studied in several protozoan parasites, including *Giardia lamblia*, *Entamoeba histolytica*, and *Toxoplasma gondii* [2]. The analysis of the repertoire of esRNAs that have been identified in these organisms has contributed to highlighting the functional specialization of the RNAi pathway, which has been suggested to participate in promoting genome stability, heterochromatin formation, and antigenic variation [2] (see below for more details).

Despite the importance of these functions, eukaryotic microbes offer some interesting exceptions to the universal presence of the RNAi mechanism: *Saccharomyces cerevisiae* and other close relative yeasts, filamentous fungi such as *Ustilago maydis* and *Cryptococcus gattii*, and protozoan parasites such as *Leishmania major*, *Trypanosoma cruzi*, and *Plasmodium falciparum*, either have noncanonical RNAi or have no RNAi pathway at all [8]–[10]. In each case, closely related microbes elaborate active RNAi machineries, suggesting that, although a very sporadic event, loss of RNAi function has occurred in several independent lineages during evolution. Given the critical role that this mechanism plays in metazoan gene regulation, the question arises whether loss of the RNAi machinery provides some evolutionary advantage that can somehow counteract the apparent disadvantage resulting from losing a mechanism that has been consolidated throughout evolution in the vast majority of eukaryotic organisms. Similarly, one might wonder about the real extent of this mechanism as a defense system against invading nucleic acids and in the regulation of biological functions in eukaryotic microbes.

## RNAi as a Defense Mechanism against Invasive Nucleic Acids

**Figure 1:**
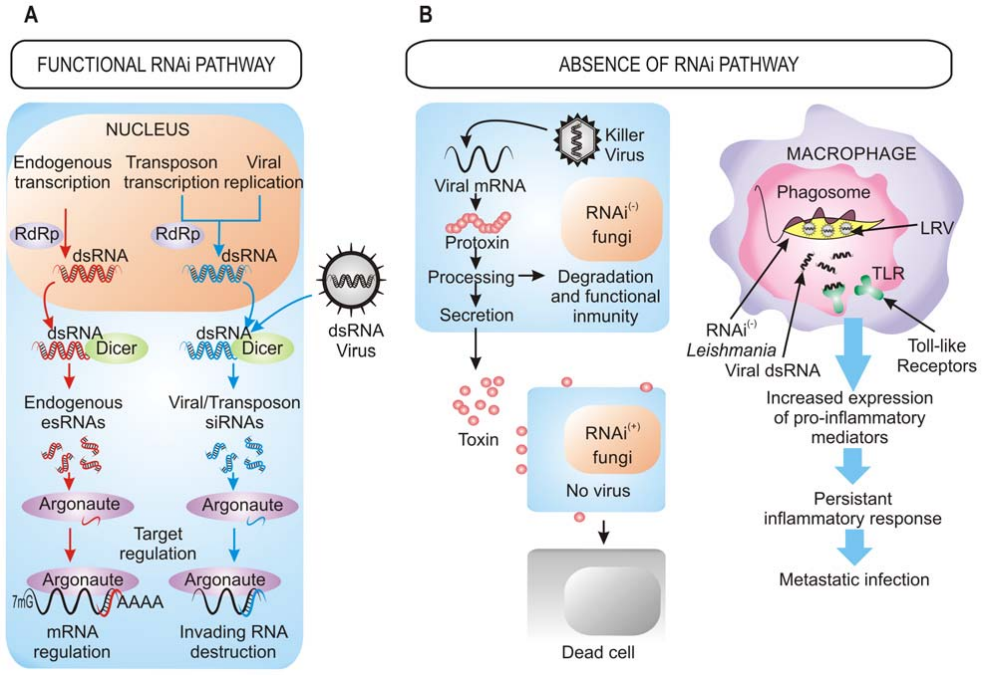
Benefits of the retention/loss of the RNAi pathway. (A) Cellular processes dependent on RNAi machinery. In organisms with a functional RNAi pathway, it plays a central role in the destruction of invading viral RNA, the elimination of transcripts from transposons, and the regulation of the expression of endogenous genes by small RNAs generated within the cell. (B) Viral pressure as a selective force for the loss of RNAi in fungi and parasites. The presence of dsRNA killer viruses in several RNAi-deficient fungi confers a selective advantage to these species over those with RNAi functional pathways (left). In *Leishmania* RNAi-deficient strains, the presence of dsRNA viruses (LRV) modifies the course of the infection, provoking an enhanced inflammatory response that results in a higher parasite burden (right). (+): functional RNAi pathways, (−): absence of RNAi pathways.

The defensive role of the RNAi pathway against exogenous nucleic acid such as viruses and transposons also operates in fungi [1] (Figure 1A). The action of RNAi against mycoviruses was first demonstrated in the chestnut blight fungus *Cryphonectria parasitica*, in which a *dicer*-like gene, *dcl2*, and an *argonaute*-like gene, *agl2*, are required for the antiviral defense response, which is based on the destruction of the viral sequences by the targeting and dicing action of the RNAi machinery [11], [12]. Mutants in any of these two genes lack the ability to avoid viral infections, becoming debilitated strains that are highly susceptible to mycovirus infections and that present hypovirulent phenotype and growth impairment once they infect their host. Similarly, *Aspergillus* mycoviruses are also targets of the RNAi pathway in *A. nidulans* [1].

The RNAi defensive role against transposons also has been demonstrated in fungi. In *N. crassa*, the RNAi pathway is able to target transposon RNAs and silence them [13], which is similar to what occurs in *Cryptococcus neoformans*, where this pathway controls transposon activity and genome integrity during vegetative growth [14]. Further approaches based in deep sequencing have also identified naturally occurring esRNAs mapping to LTR retrotransposons (LTR-siRNAs) in vegetative mycelia of the pathogenic fungi *Mucor circinelloides* and *Magnaporthe oryzae*, supporting the previously suggested role of RNAi in the maintenance of genome integrity (Figure 1A) [15], [16]. This protective role also operates during sexual development in *C. neoformans* var. *grubii*, where an increased transposition/mutation rate is detected in the progeny of crosses involving RNAi-deficient mutants [17]. Similarly, the identification of endogenous siRNAs derived from retrotransposon families in the protozoa *T. brucei*, *Leishmania braziliensis*, and *G. lamblia* suggests that one major function of the RNAi pathway in these organisms is to defend cells against parasitic nucleic acids [2]. The proposed mechanism for the RNAi pathway suggests fortuitous and aberrant transcription of the transposon, which leads to the formation of dsRNA and the consequent activation of RNAi.

## Roles of esRNAs in Fungi and Parasites

Besides defense against invasive nucleic acids, RNAi functions to regulate the physiology of the cell through the negative regulatory action of esRNAs (Figure 1A). Among fungi, in addition to the well known role of centromeric siRNAs in heterochromatin silencing, recent studies have identified different classes of esRNAs with regulatory functions, such as exonic-short-interfering RNAs (ex-siRNAs) and microRNA-like RNAs (milRNAs), isolated in *M. circinelloides* and *N. crassa*, respectively [15], [18]. milRNAs derive from single-stranded non-coding RNA transcripts with a hairpin structure and, although they regulate expression of putative target genes, their physiological relevance is still unknown [18]. ex-siRNAs derive from exons and regulate the expression of the protein coding genes from which they are produced. *M. circinelloides* mutants affected in genes involved in the production of ex-siRNAs present defects in general developmental processes such as growth and sporulation [15], which may suggest a role for these esRNAs in pathogenesis, since spore size has been identified as a virulence factor in this fungus [19]. In *M. oryzae*, esRNAs derived from tRNA fragments (tRFs) were found to be highly associated to the appressorium, a specialized hypha involved in the invasion of the host plant cell. [16]. However, a controversial question is whether the tRNA-derived small RNAs are generated by the RNAi machinery, since careful analysis of the tRNA-derived sRNAs in *T. brucei* suggests that they are degradation products [20]. This analysis shows that most of esRNAs in this parasite corresponds to retrotransposon sequences and that the majority of putative centromeric regions are devoid of siRNAs, suggesting that the main function of RNAi in *T. brucei* is the maintenance of genome integrity.

Massive sequencing of esRNAs in protozoan parasites has highlighted the functional specialization of RNAi. Besides a role in promoting genome stability, analysis of esRNAs in *G. lamblia* implicates RNAi in controlling antigenic variation and suggests a role in translation repression by miRNAs [2]. Also, in *T. gondii*, miRNAs and siRNAs molecules derived from repeated sequenced have been identified, suggesting that RNAi functions in translation regulation and heterochromatin formation [2]. The high proportion of esRNAs derived from proteins coding genes in *E. histolytica* suggests a role for RNAi in regulating gene expression [2]. Although more detailed analysis is required to determine the impact of RNAi in the biology of fungi and protozoan parasites, the emerging picture of RNAi in these organisms as a mechanism involved in a diversity of functions suggests a selective advantage to those retaining functional RNAi machinery.

## Loss of RNAi Confers Selective Advantage

The roles of RNAi described above support that RNAi is an essential mechanism that has been evolutionarily conserved through the entire eukaryotic domain. However, the inactivation of RNAi pathways by loss of *dicer*, *argonaute*, or both genes described in a number of eukaryotic microbes raises the question of how they can survive without the protection of RNAi against viruses and transposons. The answer to this question probably relies on the special evolutionary scenarios in which these species had to evolve, in which the RNAi mechanism represented a disadvantage rather than an advantage, forcing the evolution of RNAi-deficient species. This is the case of *S. cerevisiae* and other yeasts and filamentous fungi infected with “killer virus”, an endemic viral system that is cytoplasmatically inherited as dsRNA. Killer virus produces a toxin that kills nearby cells while conferring immunity to cells making the toxin (Figure 1B). Strains that retain an active RNAi mechanism process the dsRNA genome of this virus into siRNAs, losing the capability of producing the toxin and becoming susceptible to killing by toxins from cells that retain the virus [8]. Thus, the beneficial function of the RNAi as a viral defense mechanism conferred a net selective disadvantage under these circumstances. This incompatibility between the killer virus and the RNAi pathway has been proposed to explain the existence of several RNAi-deficient fungal species, including *S. cerevisiae* and other yeasts of the *sensu stricto* clade, as well as the evolutionary distant basidiomycete *U. maydis* [8]. The absence of RNAi in all sequenced *sensu stricto* yeast species while it is present in the close outgroup *S. castelli* suggests loss of RNAi in a recent *sensu stricto* ancestor, which would enable one of its descendants to acquire and retain the killer virus, providing a selective advantage over its RNAi-containing neighbors [8]. The RNAi loss has probably occurred in relatively recent times in at least nine independent fungal lineages, as suggested by phylogenetic analysis of Dicer and Argonaute proteins, which explains the discontinuous presence of RNAi in fungi [8].

The presence of dsRNA viruses as a selective force for the loss of RNAi in some trypanosomatid protozoa was first proposed in 2003 [21] and has recently been confirmed [9]. *Leishmania* species lacking a functional RNAi pathway harbor dsRNA viruses named LRVs, which have been proposed to be beneficial for the parasite by increasing survival and pathogenicity. In fact, recognition of LRVs within the *Leishmania* parasite by Toll-like receptors (TLRs) of host macrophages provokes an increased expression of pro-inflammatory molecules that renders animals more susceptible to infections [22]. Thus, the presence of the LRV virus within the pathogen subverts the immune response to infection, promoting parasite spreading and persistence that lead to a metastasizing form of leishmaniasis (Figure 1B). Loss of the RNAi pathway has been also described in other protozoan parasites, such as *P. falciparum*, which is the most virulent human malaria parasite, although the evolutionary forces leading to this loss can only be speculated [10]. However, it is interesting to note the apparent correlation between loss of the RNAi pathway and the absence of retrotransposons or viral pathogens in some protozoan parasites [10], which suggests that under these circumstances there might not be selective advantage in retaining an RNAi pathway. Phylogenetic analysis also supports the idea that RNAi genes have been lost in several independent parasite lineages during evolution [9]. The mechanisms that are responsible of the disappearance of RNAi are still unknown, although chromosomal rearrangements have been proposed to have contributed to this loss.

Lack of a functional RNAi pathway may also cause genome instability by retrotransposon rearrangements, a serious problem for most organisms that might become an advantage for a few others in certain biological contexts. This is the case of pathogenic fungi or other microbial parasites that are continuously developing new strategies to escape host defenses. In this scenario, these pathogens need a high genome plasticity and genetic diversity at the population level, which can be achieved by the active movement of retrotransposable elements. It has been speculated that *M. oryzae* utilizes LTR-siRNAs to regulate integration events of retrotransposable elements, allowing a limited transposon movement to enable, for instance, deletion of avirulence genes but ensuring global genome integrity [16]. Increased transposition activity has been also suggested as a way in which the potent pathogen *C. gattii* VGII strain R265, which lacks a functional RNAi pathway, acquired increased virulence [17]. Genome plasticity by extra-chromosomal gene amplifications has been described in RNAi-deficient *Leishmania*, where it has been associated to drug resistance. In contrast, gene amplifications are uncommon in parasites with functional RNAi pathways [9]. The differential tolerance of the RNAi-deficient or RNAi-proficient parasites to dsRNAs derived from transcription of episomal DNA may be responsible for the differential presence of amplified DNA encoding genes whose overexpression is essential for survival under drug pressure [21]. Nevertheless, although the positive effects of loss or attenuation of a functional RNAi pathway could explain the existence of RNAi-deficient species, the deleterious effects of active transposons and the lack of defenses against new evolving viruses might condemn these species to extinction over a longer evolutionary term.
